# Peptide Functionalization of Emulsion-Based Nanocarrier to Improve Uptake across Blood–Brain Barrier

**DOI:** 10.3390/pharmaceutics16081010

**Published:** 2024-07-30

**Authors:** Alberta De Capua, Raffaele Vecchione, Cinzia Sgambato, Marco Chino, Elena Lagreca, Angela Lombardi, Paolo Antonio Netti

**Affiliations:** 1 Center for Advanced Biomaterials for Health Care (CABHC), Istituto Italiano di Tecnologia, Largo Barsanti e Matteucci 53, 80125 Napoli, Italyelena.lagreca@iit.it (E.L.); paolo.netti@iit.it (P.A.N.); 2 Department of Chemical Materials and Industrial Production (DICMaPI), University of Naples Federico II, P.le Tecchio 80, 80125 Naples, Italy; 3 Department of Chemical Sciences, University of Naples Federico II, Complesso Universitario Monte S. Angelo, Via Cintia 21, 80126 Naples, Italy; marco.chino@unina.it (M.C.);; 4 Interdisciplinary Research Centre on Biomaterials (CRIB), University of Naples Federico II, P.le Tecchio 80, 80125 Naples, Italy

**Keywords:** blood–brain barrier, peptide targeting, nanoemulsions, drug delivery, transferrin receptor, glioblastoma

## Abstract

New strategies for enhancing drug delivery to the blood–brain barrier (BBB) represent a major challenge in treating cerebral diseases. Nanoemulsion-based nanocarriers represent an ideal candidate to improve drug delivery thanks to their versatility in functionalization and cargo protection. In this work, a paclitaxel-loaded nano-emulsion has been firstly functionalized and stabilized with two layers constituted of chitosan and hyaluronic acid, and, secondly, the latter has been conjugated to the CRT peptide. CRT is a bioactive peptide that selectively recognizes bEnd.3 cells, a model of the BBB, thanks to its interactions with transferrin (Tf) and its receptor (TfR). Cytotoxic results showed a 41.5% higher uptake of CRT functionalized nano-emulsion than the negative control, demonstrating the ability of this novel tool to be accumulated in brain endothelium tissue. Based upon these results, our approach can be fully generalizable to the design of multifunctional nanocarriers for delivery of therapeutic agents to the central nervous systems.

## 1. Introduction

Effective cancer therapy for the treatment of brain tumors and central nervous system (CNS) diseases remains one of the most challenging areas in drug delivery research. One of the major issues is represented by the inability to cross the physical obstacle of the blood–brain barrier (BBB) [1,2]. Identifying routes for non-invasive drug delivery to the brain and developing targeting strategies to transport biologics into the brain represent a research area of growing importance [3]. It is known from the literature that enhancing lipophilicity and positive charge is a possible strategy to increase passive diffusion, like glucose, water, amino acids and small lipophilic molecules that are crucial to neural function [4]. This is, for instance, the penetration mechanism of cell-penetrating peptides possessing multiple positive charges. However, these modifications generally lead to higher unspecific uptake in many tissues, often resulting in off-target effects since they are not selective. A promising strategy for overcoming the BBB to deliver biologics is targeting endogenous receptor-mediated transport (RMT) systems that engage vesicular trafficking to transport ligands across the BBB endothelium. Drug delivery systems (DDS), modified with appropriate targeting ligands, could improve access to the brain via RMT and release their cargo [5].

The transferrin receptor (TfR) is one of the first RMT systems studied for BBB drug delivery applications [6]. TfR is ubiquitously overexpressed on brain capillary endothelial cells because it mediates iron delivery to the brain via binding and intracellular trafficking of the iron-binding protein transferrin (Tf). Most importantly, compared to healthy brain cells, TfR has much higher expression levels in human glioblastoma because it is required for cancer cell proliferation [7,8]. The use of Tf as a targeting ligand has been demonstrated [9]. Unfortunately, there is competitive binding to TfR between the endogenous Tf and the Tf-modified DDS in vivo, thus inducing insufficient delivery to the tumor site [10]. One approach to overcome this issue is the use of targeting moieties whose TfR recognition is mediated by a different molecular pathway. A recently published disulfide-bridged cyclic peptide, CRTIGPSVC (CRT), was discovered by selection of a phage display peptide library in vivo [11]. CRT functionally mimics iron by binding to *apo*-Tf and causes the adoption of the iron-bound *holo*-Tf conformation, thereby gaining access to the brain through the Tf-TfR interaction. A CRT-labeled DDS would then be able to overcome the limitations of Tf-modified DDS by directly activating endogenous Tf towards TfR binding and internalization. This peptide exhibited promising results for the treatment of brain tumors by delivering the herpes simplex virus thymidine kinase gene to a mouse model of human glioma [11,12]. The delivery was accomplished via intravenous administration of a CRT-targeted adeno-associated virus and phage hybrid vector and resulted in significant tumor shrinkage. Another example of the therapeutic potential of CRT for glioblastoma involved the treatment with paclitaxel-loaded CRT–nanoparticles (NPs) in diseased mice, resulting in a remarkably prolonged median survival was observed [13]. Chitosan-based NPs have also been covalently modified with the widely used T7 peptide, and with the CRT-NPs have 1.9-fold higher availability by avoiding the competitive inhibition of endogenous Tf [14]. More recently, CRT functionalized core-shell nanoparticles, featuring a midazolam coating, enabled transferrin receptor (TfR)-mediated brain-targeting in mice [15].

However, although biodegradable, solid nanoparticles are classified as nanomaterials and there are some concerns regarding their use with heavy regulatory paths. Here, we propose an alternative liquid-based nanocarrier, which is an oil in water nanoemulsion (O/W NE). An O/W NE is a versatile tool in the drug delivery field, which can be easily functionalized with several targeting moieties while protecting drugs solubilized in their lipophilic phase, as in the case of solid NPs [16]. In this work, we prepare O/W NEs that are stabilized by two polymeric layers of chitosan and biotinylated hyaluronic acid, the latter being functionalized with the CRT bioactive peptide to promote the NEs’ accumulation on the BBB. An easy additive decoration strategy that exploits biotin−streptavidin physical interaction [12] is adopted to conjugate the peptide outside the system. CRT is linked to a biotinylated poly(ethylene glycol) (PEG) chain in order to inhibit the NEs’ clearance by reticuloendothelial system (RES) and expose the peptide on the external side. To verify its specificity toward cells over-expressing TfR receptor, biological tests of peptide functionalized O/W NEs have been carried out. We chose a mouse brain cell line (bEnd.3) as our model of the blood–brain barrier, and paclitaxel (PTX), a well-known cytotoxic drug, to assess peptide-mediated accumulation on bEnd.3 cells by the induced cytotoxicity. Even though PTX is an anticancer drug, its purpose in this context is mainly to assess the ability of NEs to accumulate a harmful substance toward healthy BBB cells thanks to the CRT peptide. Our results suggest that the designed vector is ready for targeted pharmacophore delivery and that further integration on the surface of a cell-penetrating peptide [17] will lead to safe crossing of the BBB by the carrier to reach the tumor site.

## 2. Materials and Methods

### 2.1. Materials

Surfactant Lipoid E80 (egg lecithin powder 80–85% enriched with phosphatidylcholine and 7–9.5% content in phosphatidylethanolamine) was purchased from Lipoid GmbH and used without further purification. Soybean oil (dietary source of long-chain triglycerides and other lipids, n_20/D_ 1.4743), ethanol (99%) and acetic acid (99.7%) were purchased from Merck. CO_2_ (≥99.998%) was purchased from Nippon gases (Italy). For preparation of all nanoemulsions and solutions, Millipore Milli-Q water was used. Chitosan (CT, LMW 90–150 kDa, degree of deacetylation (DDA) 84% determined via 1H-NMR), 1-hydroxybenzotriazole hydrate (HOBt, ≥95%), N,N′-Diisopropylcarbodiimide (DIC, 99%), N,N-Diisopropylethylamine (DIEA, 99.5%), trifluoroacetic acid (TFA, ≥99.5%, Romil), dimethyl sulfoxide (DMSO, ≥99.9%), dichloromethane (DCM, ≥99.5%), anhydrous N,N-dimethyl-formamide (DMF, 99.8%), 1,2-Ethanedithiol (EDT, 99%), Triisopropylsilane (TIS, ≥98%), piperidine (≥99%), acetone (reagent grade) and diethyl ether (≥99%) were purchased from Sigma Aldrich and used without further purification. Hyaluronic acid 250 kDa and Biotin-PEG-COOH 2kDa were purchased from Creative PEGWorks. N-α-Fmoc amino acids and coupling reagents were provided by NovaBiochem (>98%). Paclitaxel (99% purity) was purchased from Discovery Fine Chemicals Ltd, Wimborne, UK.

### 2.2. Peptide Synthesis and Purification

CRT (βA-CRTIGPSVC-βA-K) peptides were synthesized using the standard solid-phase-9-fluorenyl methoxy carbonyl (Fmoc) procedure and were obtained with good overall yields (50–60%). The syntheses were performed using a Biotage^®^Syro WaveTM peptide synthesizer (Biotage, Uppsala, Sweden). The peptide scale synthesis was 0.1 mmol. It was assembled on Rink amide resin with a substitution level of 0.71 mmol/g. The following protected amino acids were used to synthesize the peptide:

Fmoc-Lys(Boc)-OH; Fmoc-Ile-OH; Fmoc-Gly-OH; Fmoc-Ser(tBu)-OH; Fmoc-Arg(Pbf)-OH; Fmoc-Pro-OH; Fmoc-Cys(Trt)-OH; Fmoc-Ala-OH; Fmoc-Thr(tBu)-OH; Fmoc-Val-OH

The synthetic procedure can be summarized as follow:

1. Deprotection: Fmoc group was removed at the beginning of cycle with a 20% piperidine solution in DMF. After deprotection, the resin was washed with DMF to remove the residual piperidine. The peptide resin was then ready for coupling.
2. Activation: The carboxyl group of each Fmoc-amino acid was activated by addition of HBTU (2 eq.)/Oxyma Pure (2 eq.)/DIEA (4 eq.).
3. Coupling: The pre-activated Fmoc-amino acid reacted with the free amino-terminal group of the growing peptide chain on the resin using DMF as the reaction solvent
4. Capping: This reaction was performed after each coupling step, using a solution of Ac_2_O 20% and DIEA 5% in DMF. Capping cycle was introduced to prevent deletion byproducts.

Deprotection, coupling and capping steps were repeated for each subsequent amino acid, until the chain assembly was completed. When the coupling was complete, the resin was washed with DMF. At completion of the synthesis, the resin was washed several times with DMF and finally dried. The peptide was cleaved from the resin by treating it with 94% TFA/2.5% EDT/2.5% water/1% TIS for 2 h at room temperature. The mixture was then concentrated and transferred to glass centrifugal tubes for compound precipitation using ice-cold diethyl ether, which was performed repeatedly. Purified CRT peptide was obtained by preparative RP-HPLC with a Vydac C18 column (Grace, Columbia, MD, USA, 22 mm × 250 cm; 10 μm), eluted with a linear gradient (solvent A, H_2_O 0.1% TFA; solvent B, Acetonitrile, ACN 0.1% TFA) from 20 to 70% B over 58 min at a flow rate of 23 mL·min^−1^. All analyses were performed at detection wavelength of 220 nm and reported after blank chromatogram subtraction. 

### 2.3. Peptide Cyclization

CRT peptide was dissolved in an aqueous solution at a concentration of 0.1 mM. Then, DMSO was added dropwise until its final concentration was 5% (10 mL final volume). The reaction mixture was kept open to atmosphere under vigorous magnetic stirring overnight. The product was monitored by LC-MS analysis. When the reaction was completed, the water was evaporated; the peptide was precipitated in cold ethyl acetate and lyophilized.

### 2.4. Biotin-PEG-COOH Peptide Conjugation 

The peptide was conjugated at the N-term with Biotin-PEG-COOH directly on the resin. Firstly, the Fmoc protecting group was removed with a 20% piperidine solution in DMF, followed by several washing steps. Then, the coupling reaction with Biotin-PEG-COOH (2 equivalents) was conducted directly on the resin (10 mg) with DIC/HOBt/DIEA (1:1:2) 0.1 M, using DMF as solvent, overnight under nitrogen flow. At completion of the synthesis, the resin was washed several times with DMF, NMP, DCM, isopropanol and methanol, and finally dried. Biotin-PEG-peptide was cleaved from the resin by treating with 94% TFA/2.5% EDT/2.5% water/1% TIS for 2 h, precipitated in ice-cold diethyl ether and lyophilized. 

### 2.5. Peptides Analysis and Purification

The identity of crude peptides was analyzed by analytical RP-HPLC–ESI-MS. The LC-MS was performed with a Shimadzu LC-10ADvp equipped with an SPDM10Avp diode-array detector. ESI-MS spectra were recorded on a Shimadzu LC-MS-2010EV system with ESI interface and Shimadzu LC-MS solution Workstation software (ver. 3.41) was used for the data processing. A Q-array-Octapole-Quadrupole mass analyzer was used as the detector. Argon was used as ion gas in the CID cell and data were analyzed by Shimadzu LC-MS solution Workstation software. The optimized MS parameters were selected as followed: curved desolvation line (CDL) temperature 200 °C; block temperature 200 °C; probe temperature 200 °C; detector gain 1.6 kV; probe voltage +4.5 kV; CDL voltage −15 V. Nitrogen served as nebulizer gas (flow rate: 1.5 L·min^−1^). All analyses were performed with a Vydac C18 column (4.6 mm × 150 mm; 5 μm), eluted with a linear elution gradient from 1% to 70% B over 35 min at a flow rate 1 mL·min^−1^. The running eluents were: solvent A, H_2_O 0.1% TFA and solvent B, ACN 0.1% TFA.

The crude non-cyclic peptide was further purified by preparative RP-HPLC with a Vydac C18 column (22 mm × 250 cm; 10 μm), eluted with a linear gradient (solvent A, H_2_O 0.1% TFA; solvent B, ACN 0.1% TFA) from 20 to 80% B over 58 min at flow rate of 23 mL·min^−1^. All analyses were performed at detection wavelength of 220 nm. The pooled fractions, containing the desired products, were lyophilized. The peptides homogeneity was assessed by analytical HPLC and by ESI mass spectrometry. The crude Biotin-PEG-peptide was purified by preparative flash chromatography, using a Biotage ISOLERA flash purification system, ISO-1SW model, equipped with a diode-array detector. The product was eluted with a linear gradient (solvent A, H_2_O 0.1% TFA; solvent B, ACN 0.1% TFA) from 0% to 95% B over 20 column volumes, using SNAP C18 12 g as column. The pooled fractions, containing the desired products, were analyzed by analytical RP-HPLC–ESI-MS.

### 2.6. MALDI-TOF Analysis of PEGylated Peptides

PEGylated peptide was characterized by matrix-assisted laser desorption/ionization mass spectrometry coupled to two times of flight analyzers (MALDI-TOF-TOF). The sample was prepared with a final concentration of ~2 pmol/µL in the matrix by mixing the peptide with a solution 60% of α-cyano-4-hydroxycinnamic acid (CHCA) and 40% of 5-Dihydroxybenzoic acid (DHB).

The two matrix solutions were prepared as follows:

1. 20 mg/mL of CHCA in a solution of H_2_O 5% formic acid in ACN (30/70 *v*/*v*);
2. 20 mg/mL of DHB in a solution of H_2_O 0.1% TFA in ACN (30/70 *v*/*v*).

Approximately 0.25 µL of the sample was deposited on the MALDI plate after a layer deposition of a saturated solution of CHCA in acetone and allowed to dry prior to analysis. The mass spectra were recorded on an AB SCIEX TOF/TOF 5800 instrument operated in the reflector positive mode. MALDI-TOF MS analyses were conducted at a laser intensity of 4287 units and laser pulse rate of 400 Hz with a set mass range of 1000 to 6000 Da. A continuous stage motion set in a random pattern at 600 μm/s was used for sampling. Calibration was performed using Cal mix 5 from AB SCIEX as calibrants, which contained des-Arg^1^-Bradykinin, Angiotensin I, Glu^1^-Fibrinopeptide B, adrenocorticotropic hormone ACTH (1–17 clip), ACTH (18–39 clip) and ACTH (7–38 clip), resulting in a mass accuracy of 50 ppm. Each spectrum represents the sum of 2040 laser pulses from randomly chosen spots per sample position. Raw data were analyzed using TOF/TOF Series Explorer software (ver. 4.1) provided by the manufacturer and are reported as monoisotopic masses.

### 2.7. Paclitaxel-Loaded Oil in Water Nanoemulsion 

Firstly, a 20 wt% oil in water pre-emulsion was prepared. A mass of 5.8 g of lecithin Lipoid E 80 (egg lecithin powder 80–85% enriched with phosphatidyl choline and 7–9.5% content in phosphatidyl ethanolamine) was dissolved in 24 mL of soybean oil (density at 20 °C of 0.922 g·mL^−1^) at 60 °C using the immersion sonicator (Ultrasonic Processor VCX500 Sonic and Materials, Hielscher Ultrasonics GmbH Teltow, Germany), performing runs of 10 s for 1 min at 10% of sonication amplitude (microtip screwed). Then, 1 mL of ethanol solution of PTX (5 mg/mL) was added to the oil phase and kept for 1 h at 70 °C to evaporate the ethanol. Subsequently, the oil phase was added to the aqueous phase (Milli-Q water), and mixed using the immersion sonicator with runs of 10 s for 8 min at 70% of amplitude (a pulse-on and a pulse-off respectively of 10 s). The pre-emulsion was finally homogenized for 3 single cycles and 200 steps at a pressure of 2000 bar by a high-pressure homogenizer (110P series microfluidizer) to obtain the final nanoemulsion.

### 2.8. Polymers Multilayer Deposition above Paclitaxel-Loaded O/W NEs

Firstly, a layer of chitosan was deposited around the oil template with a final concentration of oil and chitosan of 10 wt% and 0.1 wt%, respectively. A 0.1 M acetic acid solution of chitosan (0.125 wt%) was prepared with a final pH = 4. Nanoemulsion 20 wt% oil was added quickly to the chitosan solution under vigorous stirring and kept under stirring for 15 min to allow uniform chitosan deposition. The nanoemulsion with the first positive layer of chitosan was passed through a high-pressure valve homogenizer at 700 bars for 100 continuous steps. The next hyaluronic acid layer was prepared by aid of two syringe pumps (HARVARD APPARATUS 11 PLUS) and an ultrasonic bath (FALC INSTRUMENTS). Starting from the secondary nanoemulsion 10 wt% oil–0.1 wt% CT, a negative charged polymer layer was deposited by mixing 1:1 (*v*:*v*) of a 0.24 wt% aqueous solution of biotinylated hyaluronic acid with the secondary nanoemulsion suspension. The two liquid phases were injected at the same flow rate (0.4 mL min^−1^) through two Polymicro flexible fused silica micrometric capillaries (inner diameter of 200 µm) interfaced at their extremities (Molex). Each drop was then collected inside a glass tube immersed in the ultrasonic bath at room temperature, 59 kHz and 100% power for 15 min. The NCs were characterized at each step of preparation by dynamic light scattering (DLS) analysis.

### 2.9. Nanocarrier Assembly

The streptavidin solution was prepared by dissolving 1 mg in 1 mL of Milli-Q water (16.6 μM). It was added to the HA-Biotin 0.12 wt%-CT 0.05 wt%-NEs 5 wt% oil, under sonication for 15 min and T = 20 °C, at a final concentration of 5.69 μM. In the same way the compound CRT-PEG-Biotin was added under sonication for 15 min and T = 20 °C to the streptavidin-HA-Biotin-CT-NEs (SAV-HA-Biotin-CT-NEs) at a molar ratio 2:1 between CRT-PEG-biotin and the streptavidin. The final concentrations were 3.2 μM and 6.4 μM for streptavidin and CRT, respectively, while the final oil weight percentage was 2.78 wt%. The NCs were characterized at each step of preparation measuring the size and Z-potential by DLS, as described next.

### 2.10. Particle Size and Z-Potential Measurements

All nanoemulsions and their successive functionalization were characterized at each step of preparation by measuring size and polydispersity index (PdI), using a Zetasizer Nano ZS device (Malvern Instruments) with a 4 mW He-Ne ion laser at the wavelength of 633 nm and a photodiode detector at an angle of 173°. All the samples were diluted to a droplet concentration of 0.025 wt% using 20 mM acetic acid at pH 4 for monolayer, and Milli-Q water for emulsions and bilayer suspensions. The calculation of the particle size distribution was performed using a default refractive index ratio (1.59) and 5 runs for each measurement (1 run lasting 100 s), at least 3 times for each sample. A particle electrophoresis instrument (Zetasizer zs nano series ZEN 3600, Malvern Instruments Ltd., Malvern, UK) was used for the Z-potential determinations. Samples were diluted as for the particle size analysis. Setting 50 runs for each measurement carried out the Z-potential analysis. Samples were collected into polystyrene cuvettes and measured three times, and the results presented are the averages of these measurements. Experiments were carried out at 25 °C. Zetasizer software 7.11 (Malvern Instruments) was used to obtain the data. Cumulate analysis was used to give the Z-average value, hydrodynamic diameter, polydispersity index and the intensity size distribution graphs.

### 2.11. Cryo-TEM Characterization

For the preparation of the frozen-hydrated sample the plunge freezing method was performed. Briefly, a drop of 3 μL of the samples were put on a previously glow-discharged 200 mesh holey carbon grids (Ted Pella, Redding, CA, USA) after that the grid was inserted in the chamber of a FEI Vitrobot Mark IV (FEI company, Eindhoven, The Netherlands) at 4 °C and 90% of humidity. The droplet of sample was blotted with filter paper for 1 s, (blot force 1, drain time 0.5 s) and then the grid was plunged into the liquid propane. The grid was then stored in liquid nitrogen in a grid box until it was finally transferred to a cryo-specimen 626 holder (Gatan, Inc., Pleasanton, CA, USA) and loaded into the cryo-transmission electron microscope for imaging. To obtain the image of the nanoparticles we used a Tecnai G2 20, a cryo-tomo transmission electron microscope (FEI company, The Netherland) equipped with LaB6 emitter (acceleration voltage of 200 kV) and recorded at with a 2 k × 2 k CCD-Eagle 2HS camera. The frozen-hydrated sample is radiation-sensitive material, so to avoid damaging; the observation was carried out in Low Dose Mode.

### 2.12. Cell Culture

bEnd.3 cells were grown in DMEM (10% FBS, 1% L-Glu, 1% streptomycin–penicillin). Cell cultures were always performed at 37 °C in 5% CO_2_ and 100% relative humidity (RH). Cells were used from passages 23 to 30.

### 2.13. Cytotoxicity Analysis

Cell viability was quantified by the PrestoBlue Assay (Invitrogen, Waltham, MA, USA) and compared to non-treated cells, which were used as a control. Briefly, 1 × 10^4^ bEnd.3 cells were seeded in a 96-well and incubated several times (30 min, 2 h and 4 h) with PTX-loaded CRT-PEG-NEs, PEG-NEs and free PTX, diluted 1:5 in cells, at a final PTX concentration of 1.4 µM. PrestoBlue Assay was performed according to the manufacturer’s procedure, after 24 h. Fluorescence of PrestoBlue reagent solution (excitation 535 nm) was read at 615 nm using a spectrofluorometer (Wallac 1420 Victor2, Perkin–Elmer, Waltham, MA, USA). All experiments were performed in triplicate. We conducted one-way ANOVA tests using MATLAB^®^ (MathWorks, Natick, MA, USA) to quantitatively evaluate cell viability at 30 min, 2 h, 4 h and 24 h.

### 2.14. Uptake of PTX-Loaded NCs 

bEnd.3 cells were cultured in DMEM supplemented with 10% FBS, 1% L-Glutamine and 1% streptomycin–penicillin. After seeding 1 × 10^4^ cells, they were incubated overnight to allow for attachment. The cells were then treated with PTX-loaded CRT-PEG-NEs and PEG-NEs, incorporating rhodaminated streptavidin during the nanoparticle assembly for 4 h at 37 °C in cell-specific medium. Post-treatment, the cells were washed twice with PBS and fixed with 4% paraformaldehyde (PAF) for 20 min. To label the nuclei and cell membranes, DRAQ5 (excitation at 633 nm) and WGA 555 were used, respectively. Fluorescence intensity was analyzed using a Zeiss LSM 700 confocal microscope equipped with a 20×/0.8NA objective lens.

Image reconstruction was performed with ImageJ. For each sample, at least 5 confocal digital images were randomly collected and analyzed. Images were acquired with a resolution of 512 × 512 pixels (949 × 949 μm). The experiment was performed in triplicates. Significant differences of data obtained from image analysis were assessed by ANOVA using MATLAB^®^ (MathWorks, Natick, MA, USA).

## 3. Results and Discussion

### 3.1. Synthesis, Purification and Characterization of CRT

**Figure 1:**
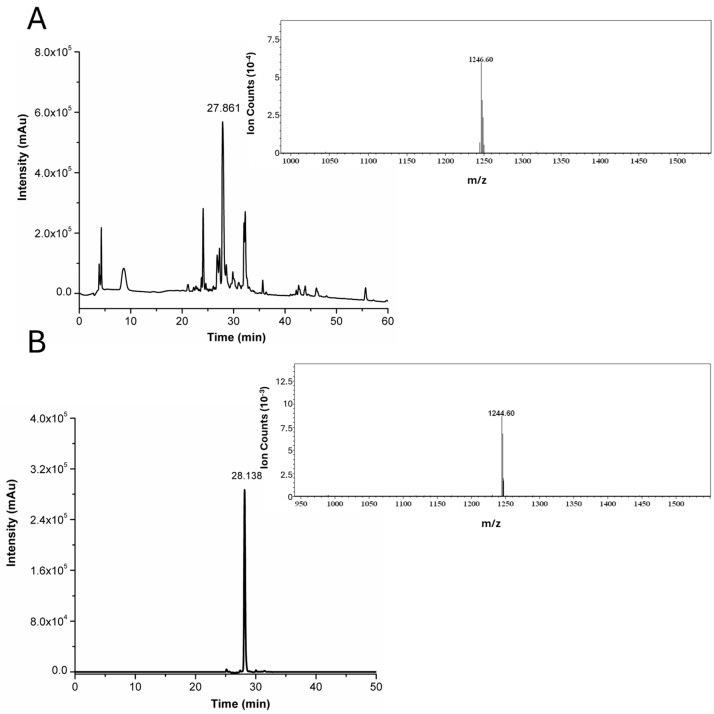
(**A**) RP-HPLC chromatogram of crude CRT. ESI-MS spectrum relative to the peak at R_t_ = 27.86 min that corresponds to [M+H]^+^ of CRT (theoretical mass: 1246.50 Da; observed mass: 1246.65 Da). (**B**) RP-HPLC chromatogram of pure cyclic CRT. ESI-MS spectrum relative to the peak at R_t_ = 28.14 min that corresponds to [M+H]^+^ of cyclic-CRT (theoretical mass: 1244.49 Da; observed mass: 1244.60 Da). Detector counts of the photodiode array are reported in arbitrary units (Au).

One of the most promising peptides that can recognize the TfR receptor is the cyclic 9-mer peptide CRT, with the sequence CRTIGPSVC. The amino acid sequence is slightly modified here by introducing a non-α-amino acid (β-alanine) at the N-term and C-term, and a lysine at the C-term as the last amino acid (βA-CRTIGPSVC-βA-K). β-alanine acts as a spacer to reduce the influence of the nanocapsule surface on the peptide conformation, while the Lys was inserted to exploit its side chain (-NH_2_) for further functionalization (i.e., fluorophore labelling). The peptide synthesis was performed using solid-phase protocols (SPPS), utilizing 9-fluorenylmethoxycarbonyl (Fmoc) chemistry and a super acid labile resin. The resulting peptide was deprotected and cleaved from the resin. The crude peptide purity was assessed by analytical RP-LC-MS, with the chromatogram and mass spectra reported in Figure 1A. The purified CRT peptide was cyclized by dissolving it in an aqueous solution containing 5% DMSO to the recommended dilute concentration of the thiol moieties (0.01–0.1 mM), thus favoring the intra-chain disulfide bond formation between the two cysteine residues over dimer formation, which is a frequent side reaction during cyclization [18,19]. Unambiguous indication of peptide cyclization was accomplished by LC-MS analysis (Figure 1B). A slight shift in the retention time of non-cyclic and cyclic peptides can be observed, followed by a two unit difference in the [M+H]^+^ values corresponding to the expected loss of two protons upon disulfide bridge formation.

**Figure 2:**
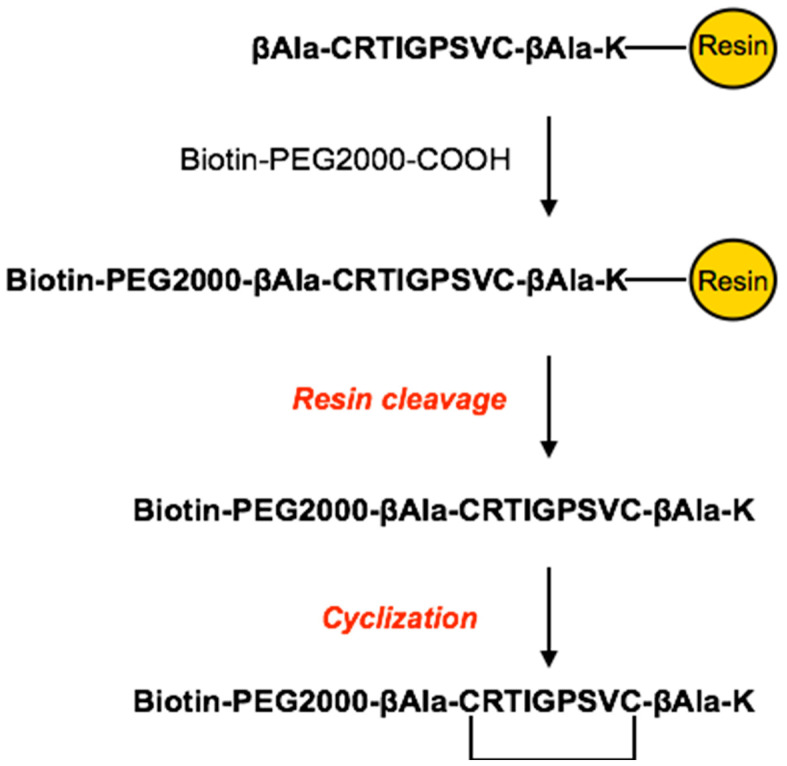
Schematic representation of the solid-phase synthetic strategy of CRT peptide PEGylation.

Peptide Biotin/PEGylation was performed to anchor the cyclic peptide to our nanocapsules by exploiting biotin–streptavidin affinity (Figure 2).

A biotin functionalized PEG linker (Biotin-PEG-COOH) was conjugated at the N-term of the peptide sequence, directly on the resin, via amide bond formation with the carboxyl group. 

**Figure 3:**
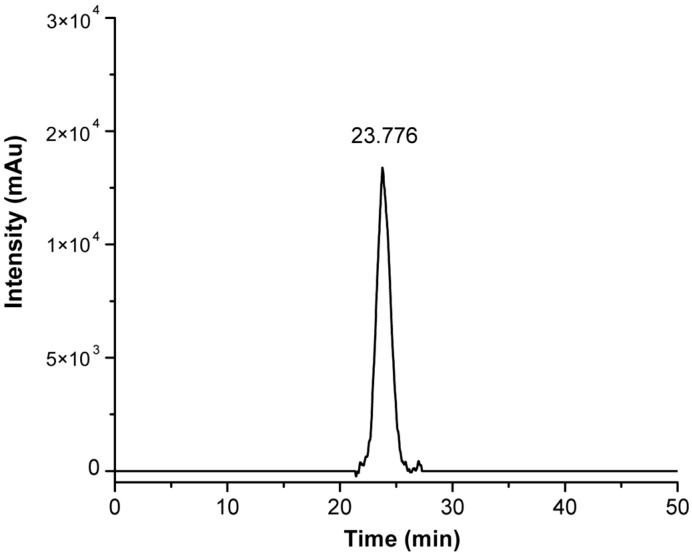
Analytical RP-HPLC chromatogram of purified CRT-PEG, detected at λ = 220 nm. Detector counts of the photodiode array are reported in arbitrary units (Au).

Solid-phase synthesis was preferred to simplify the removal of impurities from PEGylated peptide by several wash steps with a set of solvents (NMP-DCM-iPrOH-Et_2_O). Once the peptide coupling with the Biotin-PEG-COOH linker was complete, the Biotin-PEG-CRT peptide (CRT-PEG) was deprotected and cleaved from the resin. The crude CRT-PEG was purified by preparative flash chromatography, obtaining a pure product with a final yield of approximately 5%. The pooled fractions containing the desired products were analyzed by analytical RP-HPLC, with the chromatogram reported in Figure 3. 

**Figure 4:**
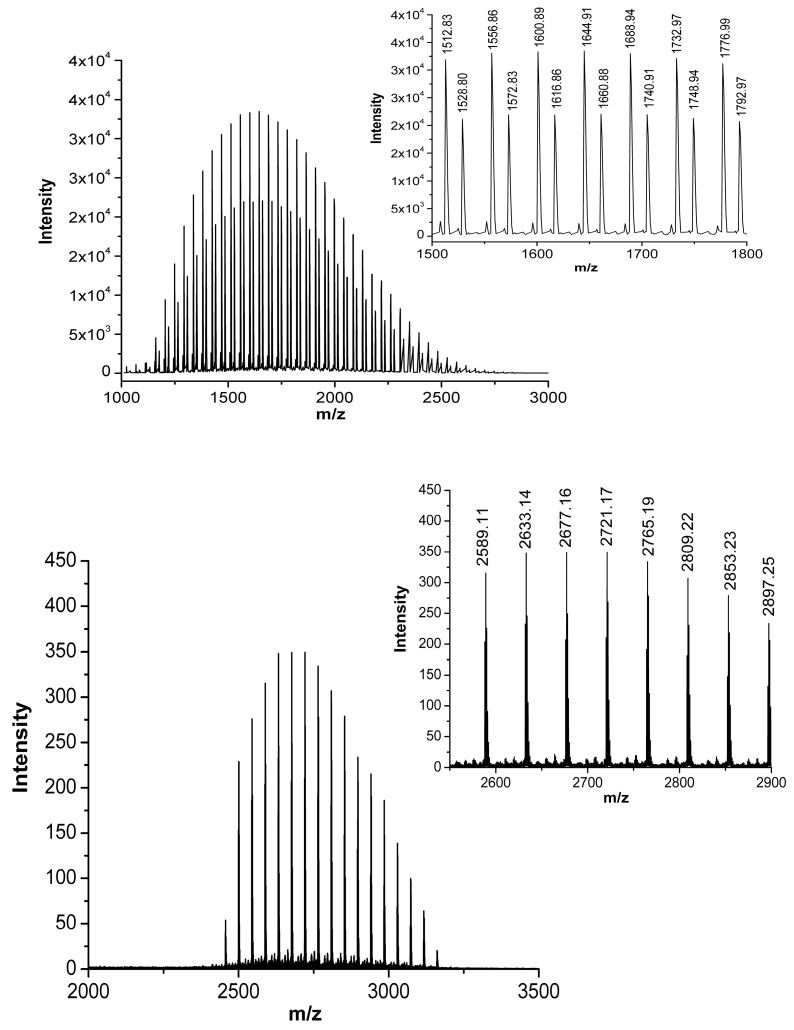
MALDI mass spectra (centroid) of Biotin-PEG-COOH linker on the **top** and CRT-PEG peptide at the **bottom**, showing the expected mass shift upon peptide conjugation. Insets report the zoomed centroid section of the spectra.

Identification of the product at R_t_ = 23.78 min was performed by matrix-assisted laser desorption/ionization mass spectrometry coupled to two time of flight analyzers (MALDI-TOF-TOF). Comparison of the centroid mass peaks of Biotin-PEG-COOH linker acquired before and after peptide conjugation confirms the reaction outcome (Figure 4).

The intense polydisperse Gaussian distribution and the typical expected ethylene oxide repeat units of 44 Da were observed, corroborating the presence of PEG within the samples. The mass increment for CRT-PEG perfectly corresponds to the CRT peptide, clear evidence that the reaction took place. The observed mass increment (2589.11 ± *n* × 44 Da) with respect to the expected theoretical isotopic mass of CRT-PEG (2566.22 ± *n* × 44 Da) is due to the +23 *m*/*z* sodiated species (MNa)^+^. Peptide cyclization was then performed as described before directly on the obtained pegylated peptide.

### 3.2. Nanocarrier Assembly 

**Figure 5:**
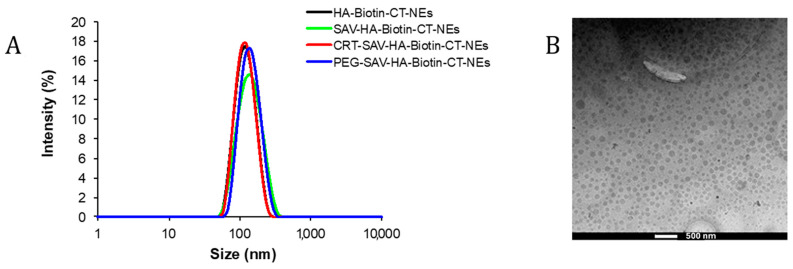
(**A**) Overlapping of hydrodynamic size of each component deposited around NEs. (**B**) Cryo-TEM projection image of paclitaxel-loaded HA-CT-NEs. Scale bar corresponds to 500 nm.

The CRT-PEG was then linked to the multi-layered NEs (CRT-PEG-NEs) to exploit its recognition of overexpressed TfR on the brain endothelium. We used a previously optimized decoration strategy [16], featuring an outer shell of biotinylated hyaluronic acid (HA-Biotin-NEs), with the only difference being that the oil core of the nanoemulsion is loaded with paclitaxel. Briefly, the streptavidin (SAV) was added to HA-Biotin-NEs under sonication, followed by the addition of CRT-PEG. The system was characterized at all stages by DLS (Table 1 and Figure 5). At all stages, narrowly monodispersed functionalized nanocapsules were obtained with a net negative charge according to the Z-potential measurements.

The morphological characterization of the paclitaxel-loaded nanocapsule was performed by cryo-TEM analysis (Figure 5B). The microscopy clearly shows a homogeneous sample fixed in its frozen hydrated state confirming the size distribution observed by DLS. The image displays a well-defined electron-dense core, corresponding to the PTX-loaded in the oil core that makes it difficult to observe the polymer layers around the O/W NEs.

**Figure 6:**
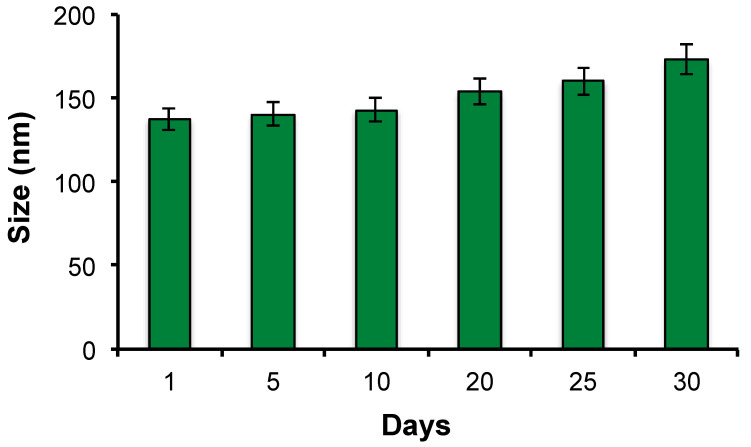
Dimensional behavior over time for PTX-loaded CRT-NEs measured by DLS analysis.

DLS periodical measurements were performed to evaluate the size evolution over time. Figure 6 shows that the hydrodynamic diameter of CRT-PEG-NEs is quite stable within the one-month detection window, with only minimal coalescence in the last 5 days (~20% radius increase).

### 3.3. Preliminary Biological Tests

bEnd.3 cells, an immortalized mouse brain endothelial cell line, represent an attractive candidate as model of the BBB due to their rapid growth, maintenance of BBB characteristics and formation of functional barriers [20]. It was demonstrated in our group that the number of TfRs per bEnd3 cells was 100-fold higher than in HUVEC cells, confirming a high value expression of TfRs [21]. To understand the best conditions to appreciate the selective activity of CRT-PEG-NEs toward bEnd.3 cells, thanks to CRT-TfRs interaction, we investigated the PTX effect on this cell line using the same procedure of Falanga et al. [22]. 

**Figure 7:**
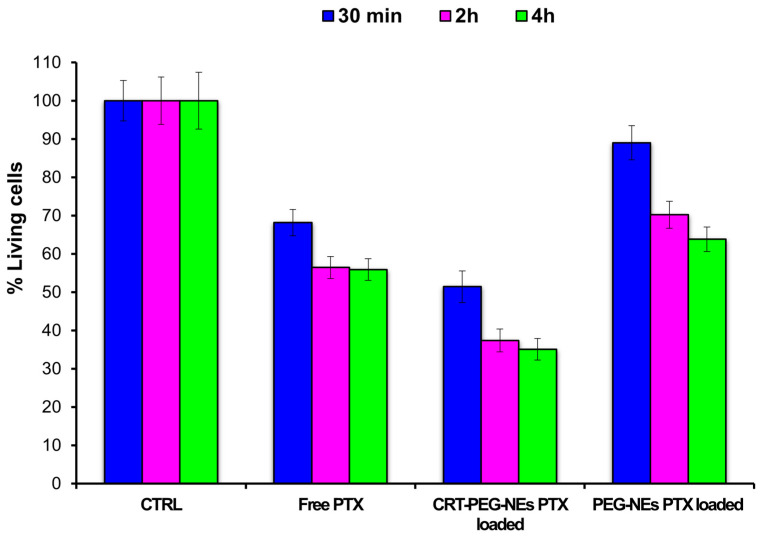
Cytotoxicity assay of PTX-loaded CRT-PEG-NEs, PEG-NEs and free PTX. bEnd.3 cells were treated at several incubation times (30 min, 2 h and 4 h) and cell viability was evaluated after 48 h. Data are reported as mean of three independent experiments (n = 3 ± SD) and expressed as percentage compared to control cells.

A confluent monolayer of bEnd.3 cells was incubated with PTX-loaded CRT-PEG-NEs, diluted 1:5 in cells, at a final PTX concentration of 1.4 µM for 30 min, 2 h and 4 h. Moreover, cells were treated with cell medium alone as positive control, free PTX as negative control and un-functionalized PEG-NEs that act as blanks. After incubation, the cells were washed and a quantitative evaluation of cell viability (normalized to positive control, which is set to 100%) was obtained by PrestoBlue^®^ assay after 48 h (Figure 7).

We conducted one-way ANOVA tests using MATLAB^®^ (MathWorks, Natick, MA, USA) to quantitatively evaluate cell viability at 30 min, 2 h, 4 h and 24 h. Data are reported as mean of three independent experiments and expressed as percentage compared to control cells. The column Prob ≥ F shows the *p*-values for the treatments (CTRL, Free drug, CRT-PEG-PXT and PEG-PXT, Prob ≥ F of 0) and time of treatments (30 min, 2 h, 4 h; Prob ≥ F of 0.0004) and the interaction between them (Prob ≥ F of 0.2525) (Table 2). These values indicate that the type and time of treatments affect the percentage yield of living cells, but there is no evidence of interaction effect of the two (Table 2).

The data showed an increase of cell mortality both for free PTX and for CRT-PEG-NEs over time, with no significant difference between two and four hours. Very interestingly, it was possible to observe a significant cytotoxicity effect of CRT-PEG-NEs compared to blank (PEG-NEs). This is an evident consequence of peptide capability to accumulate the nanocarrier on the cells surface, and allow its internalization, thanks to ligand–receptor interaction. For all the incubation times, CRT-PEG-NEs exhibit an increase of cell death relative to PEG-NEs and free PTX. For instance, at 30 min of incubation, CRT-PEG-NEs exhibit an increase of 38% of cell death relative to PEG-NEs and almost 17% relative to free PTX. Considering an average over all times, relative to un-functionalized PEG-NEs, CRT-PEG-NEs induced increased cell death of 33.05 ± 4.42%.

**Figure 8:**
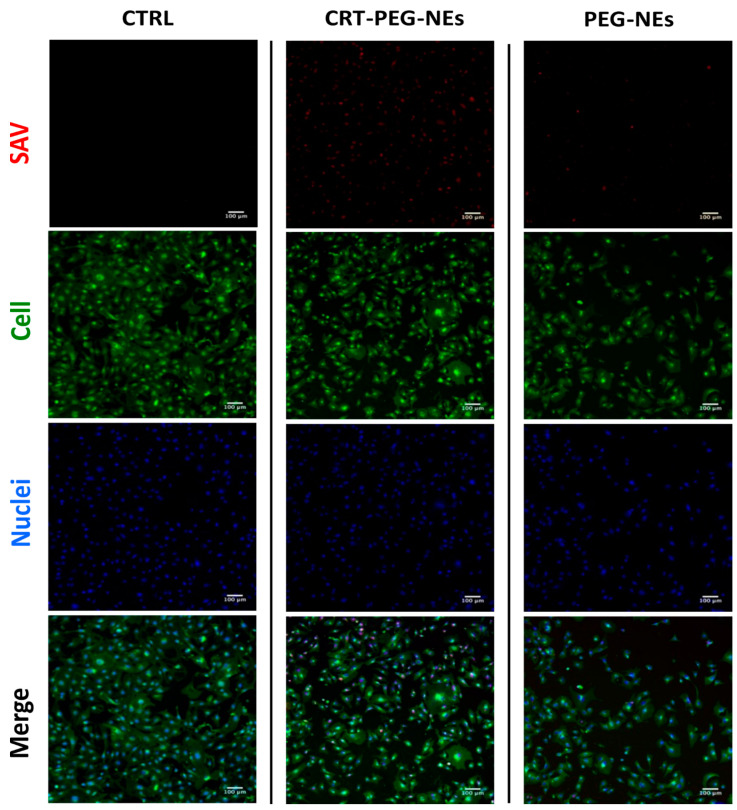
Confocal images of bEnd.3 cell. Untreated, rhodaminated CRT-PEG-NEs and PEG-NEs interactions with a confluent monolayer of bEnd.3 cells. Nuclei (blue) and cellular membrane (green) of the cells were stained with DAPI and WGA 555, respectively, while the red color represents rhodamine uptake. Scale bar is 100 µm.

**Figure 9:**
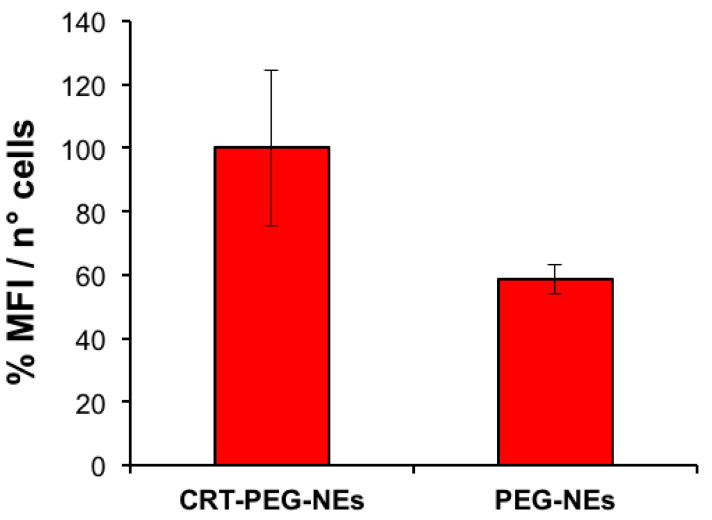
Plot of mean fluorescence intensity of rhodaminated nanocapsules normalized to cells number. bEnd.3 cells were treated with CRT-PEG-NEs and PEG-NEs. Data are reported as mean (n = 3) ± SD, *p*-value = 0.6150.

To confirm the effect due to the targeted accumulation, cellular uptake assays by confocal microscopy were performed. A confluent monolayer of bEnd.3 cells were incubated with CRT-PEG-NEs and PEG-NEs for 4 h at the same experimental condition of cytotoxicity test described before. Rhodaminated streptavidin was used during the nanocapsules assembling to detect their fluorescence. In addition, the control (CTRL) consisted in cells treated with cell medium alone. Figure 8 shows confocal microscopic images of the bEnd.3 cell monolayer after NCs uptakes, which sheds light on the previous cytotoxicity results. The presence of functionalized nanocapsules is shown in red, while the cell cytoplasm and the nuclei are in green and blue respectively. In Figure 9, the plot of rhodaminated NCs ‘mean’ fluorescence intensity normalized for the bEnd.3 cell number is reported. Particularly, three replicates for sample and at least five images for replicate are considered and statistical results are reported in Table 3. 

The uptake data are consistent with the previous cytotoxicity results, showing a clear fluorescence difference between CRT-PEG-NEs and PEG-NEs. No fluorescence was detected in the images of the control cells. Specifically, the fluorescence intensity of peptide-functionalized NCs was approximately 40% higher compared to the negative control. Furthermore, a small amount of PEG-NEs was detected in the cells. 

## 4. Conclusions

In this study, we demonstrate the versatility of engineered layer by layer nanocapsules in active targeting. To develop a novel nanocapsule able to target the BBB, our O/W NEs have been functionalized with the CRT peptide, which is able to recognize the BBB cells thanks to the Tf/TfR mechanism. O/W NEs are stabilized by a double layer of chitosan and biotinylated hyaluronic acid, and, thanks to their long shelf-life, represent an ideal candidate for further decoration of a bioactive peptide on the outer layer by an additive strategy, without purification steps. CRT-functionalized NEs preserve a narrowly distributed hydrodynamic diameter below 150 nm and are stable over time. Preliminary cytotoxic tests were carried out using a mouse brain cell line (bEnd.3) as a model of endothelial brain tissue. Results show an increased cellular death of 33.05 ± 4.42% compared to undecorated PEG-NEs that acted as a control, while the uptake of CRT-PEG-NEs increased 41.5 ± 24% with respect to the negative control. We proved in vitro the ability of the CRT peptide to target brain endothelium tissue while conjugated to the proposed O/W NE-based carrier. These in vitro results are in agreement with the current literature on CRT peptide decorate NCs. Previous strategies have utilized various nanotechnology-based delivery systems such as liposomes, polymeric nanoparticles (like PLGA NPs) and micelles. These systems often incorporate surface modifications to exploit receptors overexpressed at the BBB. For instance, PLGA NPs have been modified with transferrin receptor-targeting peptides, demonstrating improved brain targeting and reduced cytotoxicity in vitro and in vivo [23,24]. 

Recent advancements also include the use of receptor-mediated transcytosis to enhance drug delivery to the brain. Strategies such as the SGT-53 liposome, which targets the transferrin receptor, have shown promising results in glioblastoma treatment when combined with immunotherapy. Peptide-based targeting and advanced nanocarrier systems offer significant potential for improving drug delivery across the BBB, targeting tumors, and enhancing therapeutic efficacy. By leveraging passive and active targeting strategies and employing novel nanotechnologies, these approaches aim to optimize drug delivery while minimizing adverse effects. It is remarkable that here we were able to develop such an advanced functional nanocarrier starting with a simple basic nanocarrier, namely O/W NEs, thanks to the stability we can confer to it, bringing the intrinsic advantage of oil to encapsulate powerful lipophilic drugs. The proposed platform paves the way to the design of novel multifunctional nanocarriers for the delivery of therapeutic agents to the CNS with the additional advantage that the vegetable oil core is easily degraded and absorbed with no concerns in terms of accumulation in the body, in contrast with many solid-based nanomaterials. Further development will concern the integration of a cell-penetrating peptide, like gH625, able to cross the BBB. Moreover, for anticancer therapeutic purposes, potentially new therapeutics [25] or the well-established curcumin instead of PTX can be used, the latter being used in this case only as a cytotoxic molecule to test NCs’ accumulation ability. Indeed, curcumin not only has been demonstrated to be a good anticancer substance, but it is also specific for tumor cells [16]. Specifically, curcumin delivery systems that exploit the unique properties of nanoparticles and surface modifications demonstrate enhanced brain targeting and therapeutic outcomes for neurological diseases and brain tumors. The integration of these innovative delivery methods represents a promising frontier in the treatment of CNS disorders, potentially leading to more effective and safer therapies [23]. Therefore, it may be an ideal candidate for blood–brain barrier treatments, because, although a fraction of NCs will break and release curcumin in the endothelial cells instead of crossing them, such a molecule has no activity toward these cells, avoiding side effects to healthy sites.

## Figures and Tables

**Table 1:** Size, PdI and Z-potential measurement of each NCs component, loaded with paclitaxel, during the several steps of assembly. Data are reported as mean (n = 3) ± SD.

	Size (nm)	PdI	Z-Potential (mV)
NEs	89 ± 1.	0.080 ± 0.015	−27 ± 4
CT-NEs	93 ± 2	0.086 ± 0.003	+25 ± 1
HA-Biotin-CT-NEs	113 ± 9	0.094 ± 0.015	−31 ± 2
SAV-HA-Biotin-CT-NEs	131 ± 7	0.145 ± 0.017	−28 ± 3
CRT-PEG-SAV-HA-Biotin-CT-NEs	137 ± 7	0.152 ± 0.002	−30 ± 1
PEG-SAV-HA-Biotin CT-NEs	138 ± 5	0.139 ± 0.002	−30 ± 1

**Table 2:** Two-way ANOVA for cytotoxicity analysis.

Two-Way ANOVA					
Source	SS	Df	MS	F	Prob ≥ F
Columns	570.513	3	190.171	236.64	0
Rows	14.9	2	7.45	9.27	0.0004
Interaction	6.527	6	1.088	1.35	0.2525
Error	38.575	48	0.804		
Total	630.515	59			

**Table 3:** One-way ANOVA for uptake analysis.

One Way-ANOVA					
Source	SS	Df	MS	F	Prob ≥ F
Columns	2.28946e^+10^	1	2.28946e^+10^	0.3	0.6156
Error	3.09993e^+11^	4	7.74983e^+10^		
Total	3.32888e^+11^	5			

## Data Availability

Data are contained within the article.
